# Policymaker experiences with rapid response briefs to address health-system and technology questions in Uganda

**DOI:** 10.1186/s12961-017-0200-1

**Published:** 2017-05-03

**Authors:** Rhona Mijumbi-Deve, Sarah E. Rosenbaum, Andrew D. Oxman, John N. Lavis, Nelson K. Sewankambo

**Affiliations:** 1 Clinical Epidemiology Unit, College of Health Sciences, Makerere University, P.O. Box 7072, Kampala, Uganda; 2 Department of Medicine, College of Health Sciences, Makerere University, P.O. Box 7072, Kampala, Uganda; 3 Norwegian Institute of Public Health, P.O. Box 4404, Nydalen, N-0403 Oslo Norway; 4 McMaster Health Forum, Centre for Health Economics and Policy Analysis, Department of Clinical Epidemiology and Biostatistics, and Department of Political Science, McMaster University, 1280 Main St. West, MML-417, Hamilton, ON L8S 4L6 ON Canada

## Abstract

**Background:**

Health service and systems researchers have developed knowledge translation strategies to facilitate the use of reliable evidence for policy, including rapid response briefs as timely and responsive tools supporting decision making. However, little is known about users’ experience with these newer formats for presenting evidence. We sought to explore Ugandan policymakers’ experience with rapid response briefs in order to develop a format acceptable for policymakers.

**Methods:**

We used existing research regarding evidence formats for policymakers to inform the initial version of rapid response brief format. We conducted user testing with healthcare policymakers at various levels of decision making in Uganda, employing a concurrent think-aloud method, collecting data on elements including usability, usefulness, understandability, desirability, credibility and value of the document. We modified the rapid response briefs format based on the results of the user testing and sought feedback on the new format.

**Results:**

The participants generally found the format of the rapid response briefs usable, credible, desirable and of value. Participants expressed frustrations regarding several aspects of the document, including the absence of recommendations, lack of clarity about the type of document and its potential uses (especially for first time users), and a crowded front page. Participants offered conflicting feedback on preferred length of the briefs and use and placement of partner logos. Users had divided preferences for the older and newer formats.

**Conclusion:**

Although the rapid response briefs were generally found to be of value, there are major and minor frustrations impeding an optimal user experience. Areas requiring further research include how to address policymakers’ expectations of recommendations in these briefs and their optimal length.

## Background

Over a decade ago WHO made a global call for policymakers and health managers to use evidence in policies and practices to strengthen health services and systems, improve system outcomes, and achieve universal coverage and equity [1–4]. However, policymakers worldwide have encountered difficulties accessing timely and relevant research evidence of high quality [5–12].

Rapid syntheses of research findings, including rapid response briefs, are among some of the more promising strategies currently emerging to help address the challenges to, and facilitate policymakers’ use of, research evidence for policy [13–17]. Rapid syntheses are time-sensitive and can be tailored to local context, language and user needs. In this article, we define rapid response briefs as documents that present a summary of the best available evidence in a synthesised and contextualised manner in direct response to a decision-maker’s question. They are knowledge translation products that are a result of systematic and transparent methods to synthesise and appraise the evidence. They do not generate new knowledge but use already available findings, especially from existing systematic reviews [17]. Because the primary audience for rapid response briefs is policymakers, many of whom do not have a research or healthcare background, they should be short easy-to-read documents with minimal technical language, easily read and understood by very busy policymakers. However, little is known about what the optimal format of these briefs might be or if they are perceived as useful by policymakers using research evidence for policymaking.

The Ugandan country node of the Regional East African Community Health Policy Initiative (REACH-PI) [18] is a partner in WHO’s Evidence Informed Policy Network (EVIPNet) and participated in the “Supporting Use of Research Evidence (SURE) for Health Policy in African Health Systems” project [19]. Researchers piloted a rapid response service in March 2010, providing rapid response briefs on demand to policymakers in Uganda [17]. The objectives of this study were to (1) explore Ugandan policymakers’ experiences with a rapid response brief template developed for this service; (2) use our findings to improve the format of that template; and (3) assess the extent to which the revised rapid response brief template better met policymakers’ needs.

## Methods

The structure of the service, how it was developed and the process of preparing rapid response briefs have been provided in detail elsewhere [17]. The service was developed based on a literature review, brain storming, interviews with potential users and pilot testing. The process for preparing a rapid response brief includes clarifying the question with the user, ensuring that it is within the scope of the service, searching for a systematic review or, when relevant, other evidence, preparing a structured summary of the best available evidence that was found, and peer review of the brief. This process took from 1 to 28 days, depending on the urgency of the question. Examples of rapid response briefs as referred to in this study can be found in the Uganda Clearinghouse for Health Policy and Systems Research (http://chs.mak.ac.ug/uch/home).

**Fig. 1:**
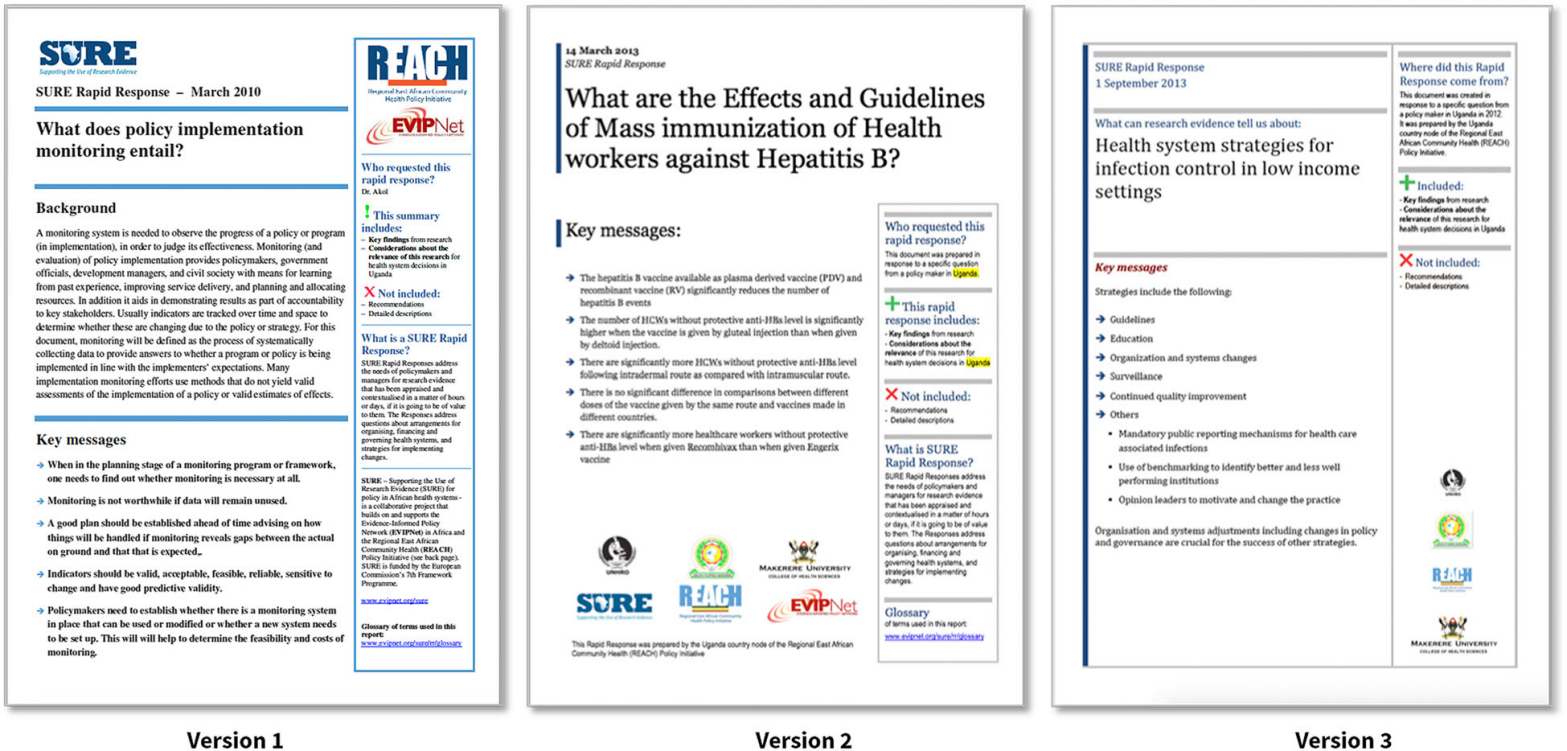
The initial format of the rapid response brief (version 1) and its two revisions (version 2 and 3)

We based the initial format of the briefs (Fig. 1, version 1) on principles for presenting evidence to policymakers learned from other studies, for example, a graded entry or layered presentation and numerical results in tables [12, 20].

We then explored user’s experiences and perceptions of the briefs using qualitative methods for data collection and analysis, and drew on these findings to agree on formatting improvements, in several cycles.

### Participants

From a list of potential public and private sector stakeholder institutions involved in or closely associated with policymaking in Uganda, we obtained a purposive sample of top and mid-level policymakers and health managers. We ensured inclusion of participants who had used the service before and those that had not. The participants were invited to take part in the user testing exercises by email or physically-delivered formal letters of invitation [21], followed by a face-to-face visit to answer any questions or concerns about the exercise.

### User testing

We explored policymakers’ experience with the rapid response brief format through a set of user tests. User testing is widely used for assessing users’ experiences with software, websites and (instructional) documents [22], including presentations of research evidence [23–27]. Representative users of a product are invited to participate in individual semi-structured interviews and asked to speak about their experience as they interact with the product [28, 29]. We adapted our methods from other studies that have user tested presentations of research evidence [20, 24–28].

### Data collection

We carried out 12 user tests iteratively between 2010 and 2013 with eight purposively sampled participants: four (out of seven invited) in 2010 using the original template and four (out of six invited) in 2012 using the second version. We then involved four participants from these eight earlier tests in a shorter session in 2013 after making changes to the second template, to check their preference between the third and the earlier template. Previous user testing has found that 80% of known usability problems could be ascertained from five representative users, with three participants revealing almost 70% of all problems including the most severe ones; there are diminishing returns after the fifth user [30–32].

In test 1, we presented users with version 1 of the template (Fig. 1, version 1), which we designed based on prior research [20]. In test 2, we presented version 2 of the template (Fig. 1, version 2), which we revised based on feedback from test 1. In test 3, we presented version 3 (Fig. 1, version 3) of the template and the version that the participant was shown in test 1 or test 2.

We carried out user testing in the form of face-to-face interviews in a quiet room, with an interviewer and a note taker, lasting approximately 1 hour and audio-recorded. We created a scenario-based interview guide designed to explore the facets of user experience as described by Morville and adapted by Rosenbaum [33, 34]. These facets, described in Table 1, include findability, usability, usefulness, accessibility, credibility, desirability and value of the product. Although Morville’s meaning of value was from the perspective of the producer, in this study we assessed value from the perspective of the user. In addition, we adopted one of the two facets Rosenbaum added to the Morville framework, that of understandability.

**Table 1:** Users’ experiences using rapid response briefs

Domain	Issues raised (illustrated with quotes where possible)					What we changed
	Major problems	Big problems or frustrations	Minor issues	Positive feedback	Specific suggestions	
Findability (the extent to which the product is navigable and the user can locate and easily find what they need)	• None	• A lot of information on the first page • Too many logos on the face/first page • Too many key messages – preference for only one or two	• Distracting information box(es) especially on the first page • A dislike for headings presented in form of questions	• Headings, colours, formatting, font are clear enough • Use of tables to summarise findings • A good balance between precision and detail of information	• Reduce information on the first/face page • Use a style that allows for citation to show in the text not at the end • More use of figures and less of text	• Only main logos left on front page, others moved to the back • Logos made smaller • No set number of key messages but instruction to keep these to a minimum • Revised what information would be on page one • Some information boxes moved to other pages
Usability (the ease of use of a product)	• None	• The report seemed lengthy • The methodology of how the report was prepared being in a small information box on the second page was easily missed • The absence of recommendations	• None	• A background section that sets the context of the report • The small size of the document • Decision options were presented clearly • The conclusion was clear • Key messages were well linked to the approaches or options provided and discussed later, which made it easy to follow • Information boxes were helpful in providing guidance on what to find in the report	• Aim to restrict text under a given subheading to the same page and not spill over to the next • Key messages should not simply be bullet points they should have a little more detail • Keep only important references	• Report should aim at 5–7 pages • Reference to the methodology to be included in the main text, in the background whenever possible (considering a balance with size of the document)
Usefulness (the extent to which the product fills a need that the user had)	• None	• The absence of recommendations or a clear way forward	• None	• All participants found the reports useful for themselves and others	• The provision of recommendations	• Communications to rapid response service team to give more time to explaining the absence of recommendations (in report and during interaction)
Accessibility (the ease with which the product is available to all users including those with different preferences or disabilities)	• None	• None	• None	• Generally found accessible “*…yeah I think, everyone, okay at least a good percentage can use this document without a problem*”	• None	• No changes made to the template
Credibility (the extent to which the users trust and believe what is presented to them and what elements of the product influence this trust)	• None	• None	• None	• Credibility attached to the research institution and partners (as identified by the logos) • Referencing using credible and trusted sources • Provision of contact information to follow up on the report and its content	• None	• No changes made to the template
Understandability (understanding (or recognising) the document category and understanding the document content)	• None	• Poor initial understanding of the type of document and its potential uses	• Unclear reference to methods used to prepare the document	• Simple language	• None	• Revision of information in the information boxes
Desirability (how much appreciation is drawn from the user by the power and value of the image, identity, brand, and other elements of emotional design)	• None	• None	• None	“*I love the report especially the background. Also it is not very long so it is readable…*” “*Very desirable, we hope this service will not end*”	• Presentation of recommendations	• No changes made to the template
Value	• None	• None	“*… the writing seems ‘laborious’. …you are spending a lot on describing yourselves…*” “*Make the reports/reports more accessible for those other than those who requested for it…*”	• None	• Increase visibility of the products through different kinds of dissemination	• Less information about partners and all of it moved to the back of the document • Decision to increase visibility with caution; need to balance demand with capacity to meet it

Using the concurrent think-aloud method [22], we provided the participants with a brief based on a topic to which we felt any policymaker could easily relate and which did not require in-depth knowledge to understand. We systematically walked the participants through the brief and urged them to comment out-loud on their experience of the different sections, highlighting any aspects they appreciated or found problematic. Although the test allowed for the interviewer to probe where necessary, any questions arising from the users during the interview were noted and only discussed after the interview to avoid distracting and biasing them.

### Data analysis

We transcribed the interviews, identifying and coding findings from the transcriptions under the following thematic areas summarised in Table 1: major problems, big problems or frustrations, and minor issues. We also looked out for positive feedback and any specific suggestions for improving the users’ experience. For the last set of user tests performed in 2013, we also identified preferences for the format before and after the changes made to the first version. All findings were coded according to their corresponding category in our adapted user experience framework. Transcription and coding was performed by the two researchers who carried out the interview, that is, the interviewer and the note taker.

The research team included three researchers from Makerere University who performed the interviews and did the initial coding and two researchers from the Norwegian Knowledge Center for the Health Services (NOKC) who participated in the latter stages of coding and thematic analysis. The entire team then discussed and came to a consensus on the themes and corrections to be made on the templates. Two senior researchers, one in Norway and one in Uganda, provided additional input during the discussions. We held the discussions through conference calls and in face-to-face meetings. The researchers at the NOKC, which included an information designer (SR), then re-designed the template based on the feedback.

This research received ethics approval from the School of Medicine, Research and Ethics Committee at Makerere University (reference number 2011–177). We sought consent for the process (including the audio-recording) from all participants.

## Results

Of the eight participants, five were from the Ministry of Health, two from civil society and one was a development partner (Table 2). The participants’ backgrounds typically included a combination of several attributes, namely medical (medical doctors and clinical officers), finance/economics, education, statistics, research, nutrition, health promotion, advocacy, government and health systems planners.

**Table 2:** Profiles of respondents involved in the user-testing exercises in this study

Initial Test No (year)	Organisation of affiliation	Type of policymaker^a^	Used service before	Participated in follow-up interview (Test 3)	Sex
Test 1 (2010)	MoH	Senior policymaker in MoH	No	No	F
Test 1 (2010)	NGO	Stakeholder in NGO/CSO	Yes	No	F
Test 1 (2010)	MoH	Policymaker in MoH	Yes	No	M
Test 1 (2010)	NGO	Stakeholder in NGO/CSO	No	No	F
Test 2 (2012)	MoH	Policymaker in MoH	No	Yes	M
Test 2 (2012)	MoH	Policymaker in MoH	No	Yes	F
Test 2 (2012)	Development partner	Development Partner, country representative	Yes	Yes	F
Test (2012)	MoH	Policymaker in MoH	Yes	Yes	M

*NGO* non-governmental organisation, *CSO* civil society organisation, *MoH* Ministry of Health

^a^Self-reported

Users’ experiences of the first version of the rapid response brief format are summarised in Table 1. The participants did not identify any major problems but identified a number of big problems including the length of the brief. One respondent felt the seven-page brief presented was too long, which reduced one’s motivation to read it. He explained his frustration:

“*…at a glance I find it* [the brief] *a bit bulky,… Usually we need policy briefs of two pages, maybe maximum five…*”

Another big problem reported was a crowded front page contributed to by what some participants felt were many key messages and various logos:

“*… the first page is like a billboard…*”

Other big problems included a poor initial understanding of the type of brief and its uses, and the absence of recommendations.

Participants also cited some minor issues and these included the fact that the information boxes on the first page were a distraction from the main text.

“*And do you have to have these* [information boxes] *here? They can’t come at the back?*”

Two participants felt there were too many information boxes on the providers of the briefs.

“*You are doing a lot of awareness of who*[m]*ever these people are …*”

Other minor issues cited included the fact that the heading had been presented as a question, and there was only implicit reference to methods used to prepare the document.

The format also had features that participants liked, such as clarity of presentation and use of tables to summarise findings.

“*…this table you see summarizes the strategies… so it will not give the reader a lot of hurdles to (pauses) yeah…*”

Participants cited a good balance between the precision and detail of information presented, a clear background section establishing the context of the brief, and the short length of the document, although the latter was a concern for two of them.

In addition, some participants felt the information boxes were in fact helpful in providing guidance on what to find in the brief. All participants found the briefs useful and expected others would feel the same. They all attached credibility to the briefs especially due to the partners represented and identified by the logos and generally found the brief usable:

“*…yeah I think, everyone, okay at least a good percentage can use this document without a problem.*”

To improve the briefs, participants suggested, among other things, that the style of referencing used be one showing references within the text. In addition, they requested the authors to consider providing recommendations and also increasing the visibility of the briefs within the target audience groups.

Participants provided conflicting feedback on three aspects of the rapid response briefs. While two participants reported the brief was long, the rest reported liking the length. Furthermore, some participants wanted additional information in the briefs while still wanting to keep them short. Five of the eight participants felt the information boxes on the right side of the page were distracting, irrelevant or misplaced. The other three felt the side boxes provided useful information about what was and was not in the brief. They also felt this information was necessary for users who have not interacted with the service or seen its products before. Three participants thought the logos on the first page were irrelevant and a form of advertising, and should be relegated to the back page. Four participants felt the logos gave credibility to the brief.

We attempted to balance how we addressed the different concerns in the revised template. For example, concerning the length of the brief versus the information useful for understanding the document, we maintained the guidance that the briefs should be limited to five pages and attempted to provide additional information online or through personal communication. Further, we kept the side boxes, while reducing the amount of information in them and emphasising key information, and we kept the logos on the first page, while reducing their size.

### Overall preference for version 2 and version 3 of the templates

Two of the participants preferred the revised template while two preferred the older one (Table 3).

**Table 3:** Users’ preferences for the alternative versions of the revised rapid response brief

Which participant	Preference	Explanation why (illustrated with quotes if possible)	User experience category
Respondent 1	Version 3	Less crowded face/first page	Findability
Respondent 2	Version 2	Version 3 template looks “*deficient*” and still has no recommendations	Usability
Respondent 3	Version 2	Version 2 template is fine so long as you keep the document short	Usability
Respondent 4	Version 3	Face page is more attractive – makes the document feel “*light*”	Findability

The reasons cited for preference of version 3 of the template were that the face page was less crowded and made the document feel ‘light’ and attractive. Those participants preferring the older version 2 did not have particular reasons. They said that the newer template looked ‘deficient’, but did not have any new recommendations. One respondent reported that there was no need for changes as long as the document was kept short. Although preferences for the different versions differed, none of the participants felt there were still any big problems with the final version (version 3).

## Discussion

This study explored Ugandan policymakers’ experiences with a rapid response brief format and the extent to which subsequent changes to the template improved their experiences. Although we did not uncover any major problems, there were several large problems, causing confusion or difficulty, which were ultimately resolved to some extent. In addition, there was positive feedback and suggestions from participants.

### Findings in relation to other studies

#### Problems

A large problem reported by one participant in this study was the length of the brief, a finding previously reported by others [12, 20, 35]. It is uncertain what the optimal number of pages is for such a document, but several studies have shown that policymakers do not take the time to read lengthy reports, and have a clear preference for short and concise reports or summaries of research [12, 20, 35]. Rapid response briefs are meant to help policymakers in urgent situations and, therefore, limited by time. Scientists and researchers are thought to have more available time to read longer reports, whereas policymakers may not [36–38].

Another big problem was the absence of recommendations in the brief. This frustrated all but one of the participants. Policymakers have expressed similar frustrations with SUPPORT summaries of systematic reviews [20] and evidence briefs for policy [39]. Moat et al. [40] found that “*not concluding with recommendations*” was the least helpful feature of these briefs. There are many reasons for which we did not include recommendations. Rapid response briefs seek to summarise relevant research evidence and may not incorporate other relevant information or consider all of the factors relevant for a decision. Furthermore, recommendations do not flow directly from research evidence. They reflect the judgments, views and values of the authors. Although recommendations might be within the scope of evidence briefs aiming to fully address the pros and cons of different options for addressing a problem [41], they are clearly outside of the scope of rapid response briefs, which aim to summarise succinctly the research evidence addressing a specific question. Therefore, it is necessary to find ways of clearly communicating to policymakers the purpose of rapid response briefs and to ensure they know what to expect in them.

Another major problem participants cited was a crowded first page because of multiple logos, multiple key messages and information boxes. Indeed, anecdotal evidence shows crowded text, lots of images, multiple font styles, and too much information can make a page very difficult to read [42]. Furthermore, there was confusion about what kind of document the brief was and how it had been prepared despite the information boxes describing this. This may reflect the fact that participants did not read the information boxes. Some expressed that these boxes were a distraction from the main content. It is also possible that participants read the information in the boxes but did not find it clear or sufficient. A potential solution would be to include a methods section in the text, but this would need to be balanced with concerns about the length of the brief. More interaction between users of the briefs and researchers might also help users to understand the methods used to prepare the briefs. Links to additional information online might be another way to provide this information without increasing the length of the briefs.

A minor issue cited was the feeling that the briefs were not visible to others who would potentially benefit from them. Since the research team deliberately took measures to limit the demand for services during the pilot, this may have limited its visibility. We expected visibility to improve after the pilot period.

#### Positive feedback

We found participants valued the rapid response briefs and found them to be useful. This is in keeping with findings from a similar study exploring policymakers’ experiences with a short summary of results of systematic reviews relevant to low- and middle-income countries [20].

One feature the participants felt increased the credibility of the briefs was the use of references from sources in which participants already had confidence, for example, well-known journals or The Cochrane Library. Rosenbaum et al. [20] also found the use of trusted sources increased the credibility of research summaries.

Considering the templates as a whole, there was equal preference for the two versions. However, this preference was not tagged to any major frustrations or big problems, but to guidance that can be adopted for either version; for example, the need to keep the brief short and not to crowd the front page.

Participants provided conflicting feedback about several aspects of the rapid response briefs. It is important to note that user testing does not look for consensus but welcomes all feedback to enable consideration of all potential difficulties from would-be users. It is possible the conflicting feedback seen in this study reflects differences in the backgrounds of the participants; for example, their training in research methods, level in the policymaking hierarchy, prior exposure to rapid response briefs, or exposure to other types of evidence products. It would be difficult, if not impossible, to tailor the format of the briefs to such differences although we attempted to balance how we addressed these concerns in the revised template.

### Strengths and limitations

As far as we are aware, this is the only study that has evaluated users’ experience of reports or briefs of rapid responses to policymakers’ needs for research evidence. We used a concurrent think-aloud protocol widely used for user testing, which has been used in the past for evaluating how users experience the format of different types of evidence-based reports [23, 41, 43]. This approach avoided the risk of recall bias, which might have occurred if we had used a retrospective approach in which participants have to think back on their experience. In addition, the concurrent think-aloud approach can reveal more unspoken feedback than a retrospective approach. Although the need to think aloud while working can have a negative effect on the task being performed, and could affect participants’ perceptions of the briefs, it is unlikely this had a major impact on our findings, since the task of perusing and reading documents is one the participants perform regularly.

In this study, we involved eight participants (four for each version of the template), a number higher than that thought to reveal 80% of known usability problems, including the most severe ones. We believe we were able to capture most problems associated with rapid response briefs, especially the major ones.

Another strength of this study is that we did not only use the feedback from the user testing to make changes to the template. We conducted follow-up interviews after these changes to ensure there were no new major problems created or old ones left unhandled.

An important limitation of this study was the ‘lab’-like context. Rather than observing people using these briefs in an actual decision-making process, we invited them to interviews in which we designed policymaking scenarios which were not necessarily relevant for each of the participants. However, the briefs provided were those requested from actual policymaking processes and the topics of the briefs chosen were ones to which most policymakers would be able to relate.

Another potential limitation of this study is that fewer participants engaged in the third test where we tried to gauge preference for the two revised formats. Four participants might be considered too few to give a representative result. However, the aim of user testing is not primarily to create generalizable findings but to identify potentially important problems and experiences from people who represent the target group, and this was achieved.

### Implications for practice and research

The main findings of this study are that users of rapid response briefs value them and for the most part, view the format of the briefs positively. The rapid response team in Uganda now uses the final template (version 3). An important challenge that should be addressed in practice and further research is balancing users’ preference for short briefs with their desire for more information. In addition, further user testing in other contexts, testing using actual scenarios and comparative evaluations of the effects of the rapid response service on decisions and other outcomes are needed.

## Conclusion

Policymakers in Uganda found rapid response briefs to be useful, accessible and credible. However, they experienced some frustrations and there was some conflicting feedback, including different views about the length of the briefs, presentation of information about what the briefs are and are not and how they were prepared, and the absence of recommendations.
